# Development of Policy Relevant Human Biomonitoring Indicators for Chemical Exposure in the European Population

**DOI:** 10.3390/ijerph15102085

**Published:** 2018-09-21

**Authors:** Jurgen Buekers, Madlen David, Gudrun Koppen, Jos Bessems, Martin Scheringer, Erik Lebret, Denis Sarigiannis, Marike Kolossa-Gehring, Marika Berglund, Greet Schoeters, Xenia Trier

**Affiliations:** 1 Flemish Institute for Technological Research (VITO)—Sustainable Health, 2400 Mol, Belgium; gudrun.koppen@vito.be (G.K.); jos.bessems@vito.be (J.B.); greet.schoeters@vito.be (G.S.); 2 German Environment Agency (UBA), 14195 Berlin, Germany; Madlen.David@uba.de (M.D.); marike.kolossa@uba.de (M.K.-G.); 3 Department of Chemistry and Applied Biosciences, Eidgenössische Technische Hochschule (ETH), 8092 Zürich, Switzerland; scheringer@chem.ethz.ch; 4 Research Centre for Toxic Compounds in the Environment (RECETOX), Masaryk University, 611 37 Brno, Czech Republic; 5 Institute of Risk Assessment Sciences (IRAS), Utrecht University, 3508 TC Utrecht, The Netherlands; erik.lebret@rivm.nl; 6 Department of Chemical Engineering, Aristotle University of Thessaloniki, 54124 Thessaloniki, Greece; d.a.sarigiannis@gmail.com; 7 Institute of Environmental Medicine (IMM), Karolinska Institutet (KI), 171 77 Stockholm, Sweden; marika.berglund@ki.se; 8 European Environment Agency (EEA), 1050 Copenhagen, Denmark; Xenia.Trier@eea.europa.eu

**Keywords:** indicator, human biomonitoring, HBM, science-policy translation, HBM4EU, health-based guidance value, groups of substances

## Abstract

The European Union’s 7th Environmental Action Programme (EAP) aims to assess and minimize environmental health risks from the use of hazardous chemicals by 2020. From this angle, policy questions like whether an implemented policy to reduce chemical exposure has had an effect over time, whether the health of people in specific regions or subpopulations is at risk, or whether the body burden of chemical substances (the internal exposure) varies with, for example, time, country, sex, age, or socio-economic status, need to be answered. Indicators can help to synthesize complex scientific information into a few key descriptors with the purpose of providing an answer to a non-expert audience. Human biomonitoring (HBM) indicators at the European Union (EU) level are unfortunately lacking. Within the Horizon2020 European Human Biomonitoring project HBM4EU, an approach to develop European HBM indicators was worked out. To learn from and ensure interoperability with other European indicators, 15 experts from the HBM4EU project (German Umweltbundesamt (UBA), Flemish research institute VITO, University of Antwerp, European Environment Agency (EEA)), and the World Health Organization (WHO), European Core Health Indicator initiative (ECHI), Eurostat, Swiss ETH Zurich and the Czech environmental institute CENIA, joined and contributed to a workshop, held in June 2017 at the EEA in Copenhagen. First, selection criteria were defined to evaluate when and if results of internal chemical exposure measured by HBM, need to be translated into a European HBM-based indicator. Two main aspects are the HBM indicator’s relevance for policy, society, health, and the quality of the biomarker data (availability, comparability, ease of interpretation). Secondly, an approach for the calculation of the indicators was designed. Two types of indicators were proposed: (‘sum) indicator(s) of internal exposure’ derived directly from HBM biomarker concentrations and ‘indicators for health risk’, comparing HBM concentrations to HBM health-based guidance values (HBM HBGVs). In the latter case, both the percentage of the studied population exceeding the HBM HBGVs (PE) and the extent of exceedance (EE), calculated as the population’s exposure level divided by the HBM HBGV, can be calculated. These indicators were applied to two examples of hazardous chemicals: bisphenol A (BPA) and per- and polyfluoroalkyl substances (PFASs), which both have high policy and societal relevance and for which high quality published data were available (DEMOCOPHES, Swedish monitoring campaign). European HBM indicators help to summarize internal exposure to chemical substances among the European population and communicate to what degree environmental policies are successful in keeping internal exposures sufficiently low. The main aim of HBM indicators is to allow follow-up of chemical safety in Europe.

## 1. Introduction

To inform the safe management of chemical substances in Europe and hence protect human health against chemicals related adverse health effects, 27 countries are currently collaborating in the human biomonitoring initiative HBM4EU (Human Biomonitoring for Europe) [1]. HBM4EU is a Horizon2020 Framework Project, which started as a European Joint Project (EJP) for the development of a sustainable European wide HBM network (2017–2021). Human biomonitoring (HBM) measures the presence of exogenous chemicals in human samples such as urine, blood, or hair. The analytes may be parent compounds and/or their metabolised transformation products (metabolites). These analytes are called exposure biomarkers. HBM estimates the internal chemical exposure or body burden and shows that the chemicals of interest are taken up (absorbed) by the body. Internal concentrations may stem from multiple sources, and the resulting internal exposure is thus reflective of aggregate exposure. 

HBM4EU aims to coordinate and advance human biomonitoring in Europe [2]. HBM4EU also aims to support policy making by providing better evidence of the actual exposure of citizens to chemicals and allow for the interpretation of how exposure biomarkers may relate to health effects. The purpose of HM4EU is to inform current and future environmental policies and strategies related to chemicals, environment, and human health. This includes both existing European Union (EU) and national chemical policies as well as future actions highlighted in the EU 7th Environmental Action Programme (EAP), with the overall aim to assess and minimise human health risks from the use of hazardous substances by 2020 [3,4,5]. HBM4EU will: (i) harmonise procedures and tools for HBM at EU level; (ii) provide access to and, where missing, generate internal exposure data and link this data to (aggregate) external exposure and the relevant exposure pathways; (iii) develop novel methods to identify human internal exposure to, amongst others, environmental and occupational chemicals and establish the causal links with human health effects; (iv) provide policy makers and the general public with science-based knowledge on the health risks associated with exposure to chemicals; and (v) improve chemical risk assessment in the EU through the effective use of HBM data. The research results should be targeted, timely, and fit for purpose. The major goals are effective science-policy translation and communication to policy makers responsible for assessing and managing the risks to human health from chemical exposure via the environment, food, consumer products, and occupational activities. Scoping reviews for groups of substances prioritized within HBM4EU were developed. These contain information on policy questions and available internal levels measured in the European population via HBM, health effects, exposure characteristics, and regulatory context. Policy makers at both national and EU levels provided input for prioritisation of the chemicals and formulated policy questions to allow HBM4EU research to be targeted to the policy needs. Key societal and political questions were how chemicals may impact our health, what their sources are, if sub-populations or inhabitants of specific geographic regions are impacted differently, and how policies or other risk management options may be designed to prevent risk from chemicals. HBM indicators, which can help to answer these questions, were lacking. Therefore, a method for the construction of HBM indicators was developed. 

Indicators are tools to illustrate complex scientific information using a few carefully selected variables, and thereby communicate the main messages to a non-expert audience [6]. They serve multiple purposes: They can guide policy makers to take actions in order to achieve goals set by policies or demanded by society, they facilitate the measurement of distances to targets, they have a signalling function, and they help raise awareness across different stakeholder groups [7,8]. 

Human biomonitoring (HBM) is situated at the intersection of environment and human health [9]. Environmental health indicators aim at building a core knowledge on a continuum from source to exposure to health outcome. HBM data are positioned close to the impact level in the pressure-state-impact response framework, as they represent actual exposure and the result of uptake (absorption) and disposition (fate in a human’s body following absorption) of the chemicals and are used as scientifically most-justified dose-estimates in exposure–response relationships that link exposure to (potential) health outcomes.

Indicators are influenced by the value judgement on the question they are designed to provide an answer to. The value judgement is not objective but is related to societal norms of what is ‘desirable’ or ‘undesirable’, which may be affected by political or cultural preferences and related ethical reasoning. In relation to the value judgement implied in the question asked (or the set goals), an indicator ideally has to have a transparent connection with a normative statement [10]. Such a statement, which speaks about ‘desirable’ and ‘undesirable’ states of the system, needs to be supported by a reasoning that explains why a certain state is more desirable than another. 

Presenting HBM data in the form of an indicator informs strategy and policy development in the area of chemicals management on the one hand, while at the same time, it may serve to check progress towards policy targets already set (normative statements). HBM indicators may provide valuable information on the progress of national and international activities on environment and health, including those led by the European Parliament, the European Commission (EC), OECD (Organisation for Economic Co-operation and Development), and WHO. They may also provide knowledge on the progress on the UN (United Nations) sustainable development goals (SDGs) that have integrated health as an outcome of sustainable development goals no. 3 (good health and well-being) [11], no. 6 (clean water and sanitation), no. 10 (reduced inequalities), and no. 12 (responsible consumption and production) (see Table S1 in supplementary material for some examples). Their objectives differ, but the protection of human health is a common and primary goal. Indeed, human health and safety is an important driver under the horizontal EU chemicals legislation REACH (Registration, Evaluation, Authorization and Restriction of Chemicals), and is also a priority for the OECD Environment Directorate within the context of sustainable development of regions [12]. In the EU, HBM indicators could also be a tool to assess the 7th Environmental Action Programmes objective of creating a non-toxic environment in the complicated context of a circular economy, allowing the overall efficiency of existing limits and restrictions to be evaluated, and exposures to both currently used chemicals and legacy chemicals, as well as their substitute chemicals, to be surveyed.

The terminology of indicators varies in the fields in which they are applied. Eurostat differentiates between ‘result’ and ‘impact’ indicators. Measurement of the concentration of a substance in blood or urine could be defined as ‘HBM indicator of internal exposure’, and is an example of a result indicator. It does not give information on the hazard of the chemical, at which level an effect will occur (potency) or of the risk. Changes of internal exposure levels with time, age, sex, and geographical location can be evaluated. Furthermore, a comparison can be made between HBM values of specific sub-populations on the one hand and the so-called HBM reference values (HBM RV) representing the high-end (mostly the P_90_ or the P_95_) of the internal exposure levels (HBM values) of a representative sample of individuals from the general population. It highlights exposure in specific subpopulations vs. the population as a whole. Monitoring trends in human chemical exposure can be related to chemicals in environmental compartments, food, or consumer products. This ‘result’ indicator can be compared with a level below which no adverse health effect is expected (for example, the HBM health-based guidance value or HBM HBGV). In that case, a judgement is made on the ‘Margin of Internal Exposure’ (i.e., the ratio of the HBM result indicator to a so-called “safe internal exposure level” based on Health-based guidance values) and it can be named a ‘HBM indicator for health risk’.

The present manuscript describes the process used in HBM4EU to develop indicators and is illustrated by three examples of actually constructing them.

## 2. Methods 

### 2.1. Process for Indicator Development

To ensure interoperability with other indicators and initiatives, both within HBM4EU, Europe, and globally, a consultation round with experts in the development of the HBM4EU indicator methodology was organised. This was done during an expert workshop held in June 2017 in Copenhagen at the EEA. The workshop was attended by 15 experts, including researchers of the HBM4EU project (EEA, VITO, UBA, University of Antwerp), as well as experts involved in indicator development from Eurostat, European Core Health Indicators (ECHI), World Health Organization (WHO) Europe, the World Health Organization (WHO), Swiss ETH Zurich and the Czech environmental institute CENIA. The following items were on the agenda: (i) to discuss and receive inputs on criteria for selecting the HBM4EU indicators and on calculation methods; (ii) to discuss the possible use of HBM indicators in relation to the policy targets they could support; (iii) to get an overview of how other chemical indicators are developed and which objectives they support; (iv) to discuss how the indicators support the communication to various target audiences. Each of the participants presented examples, methodologies, and reflections on indicators that were developed within their domains. After the workshop, a manuscript was prepared and work package leaders of HBM4EU responsible for work packages on mixtures and on modelling were invited to comment. 

### 2.2. Building HBM Indicators

#### 2.2.1. Selection of Substances as HBM Indicators

Selection criteria were defined to evaluate if there was a need to inform on specific HBM results by means of a European HBM indicator. The criteria were discussed in the workshop. The selection criteria were partly based on those used by: ECHI, the Norman Networks indicators for chemicals in water, and the environmental indicators of the EEA.

#### 2.2.2. HBM Data Used for Calculation of HBM Indicators 

Two chemical substance groups (being handled within HBM4EU) were chosen as a case study. Grouping of substances in HBM4EU was considered if: (i) common analytical methods can be used to analyse multiple substances in one matrix; (ii) the substances have similar uses, with the possibility of substitution within the group; (iii) the substances have a similar toxicological profile. For the bisphenols priority group, bisphenol A (BPA) was selected as the lead substance. For the per- and polyfluorinated alkyl substances (PFASs), the following lead substances were selected: perfluorooctanesulfonic acid (PFOS), perfluorooctanoic acid (PFOA), perfluorononanoic acid (PFNA), and perfluorohexane sulfonate (PFHxS). 

The BPA data used in this manuscript were published data from the EU FP-7 and Life + project DEMOCOPHES. It was a European-wide HBM pilot study in which urine samples from children and their mothers were sampled in 17 European countries (EU-FP7) (*N* = 1844). Samples were collected in 2011. Urinary BPA was analysed after enzymatic hydrolysis in samples of mothers (<45 years old) and their children (5–12 years old) (*N* = 639) from only six European countries [13,14]. The numbers of participants per country are given in Table S2 (supplementary material). The PFASs (PFOA and PFOS) were measured in serum and were only available for the Danish DEMOCOPHES participants [15]). Additionally, Swedish data were used to illustrate time trends. Since 1996, Sweden has run a health-related environmental monitoring programme in which they have measured PFAS, belonging to the groups of perfluorocarboxylic acids and perfluorosulfonic acids, in children, young men, and women for various years [16,17,18]. Data were aggregated from these publicly available studies. 

#### 2.2.3. HBM Health-Based Guidance Values (HBGVs) 

To illustrate the indicators, widely accepted health-based guidance values such as the HBM-I value from the German Human Biomonitoring Commission [19] were used to evaluate health risks of the study population. The HBM-I value represents the concentration of a substance in human biological material below which, based on the current state of knowledge, there is no risk of adverse health effects. HBM HBGVs for BPA in urine are 100 µg/L for children and 200 µg/L for adults [20]. HBM HBGVs for PFOS and PFOA in blood are 2 µg PFOA/L and 5 µg PFOS/L in plasma [21]. No HBM-I value for PFNA is available. Also, despite PFHxS being classified as a substance of very high concern (SVHC) (i.e., a very persistent and very bioaccumulative (vPvB) substance), no HBM HBGV is currently available in Europe or elsewhere.

#### 2.2.4. Method for Calculation of HBM Indicator

***HBM Indicator of Internal Exposure***

Indicators often use available aggregated data, such as summary statistics of measurements (e.g., P_50_). For single substances, P_50_ and P_95_ values were used to calculate HBM indicators representing exposure levels in the whole population, or stratified parts of the population (according to age, sex, Socio-Economic Status (SES)). 

To calculate an indicator for a group of substances at population level, the P_50_ values were used, though the P_95_ or some other P_x_ also could be used:

Sum exposure = P_x, SUBSTANCE, 1_ + P_x, SUBSTANCE, 2_ + …. + P_x, SUBSTANCE, *n*_ (1)

where P_x_,_SUBSTANCE_,*_n_* is the x’th percentile of the HBM concentrations of the n’th substance at population level. Identical type of indicators are used by US EPA (United States Environmental Protection Agency) for PCBs (polychlorinated biphenyls) and PBDEs (polybrominated diphenyl ethers) [22]. In the US, it is recommended to group only substances that have similar structural or functional properties, if they can substitute each other or have similar toxicological profiles.

In the current paper, another grouping variant was also used in which the concentrations of perfluorinated alkyl acids (PFAAs) being a subgroup of the larger group of PFASs, were summed per year in each of the age categories in which it was measured. The average result indicator was then calculated by taking a simple average of the age groups. Weighting factors, taking into account the age distribution of the whole population, were not applied, since it would introduce a bias on the P_x_ value. 

***HBM Indicator for Health Risk***

Two other types of HBM indicators were calculated, intrinsically including an evaluation against HBM HBGVs. The first one is the ’Percentage of population (i.e., individuals) exceeding the HBM HBGV (called PE), and the second is the on-population level ’Extent of exceedance’ (called EE). The percentage of the population exceeding the HBM HBGV is given as:

PE = *n*/*N* × 100% (2)

with *n* being the number of samples that have HBM-exposure levels above the HBM HBGV and *N* being the total number of samples. 

The extent of exceedance (EE) describes the extent by which the HBM HBGV is exceeded. It is calculated by dividing the P_x_ percentile by the HBM HBGV:

EE = P_x_/HBM HBGV (3)

A value >1 means that for the percentile P_x_ the HBM HBGV is exceeded. Below 1, there is no exceedance for the P_x_.

Consensus was found in calculating the extent of exceedance indicator (EE) of single substances for P_50_ and P_95_. Whatever P_x_ is selected for use in the extent of exceedance indicator, more information on how the distribution of the HBM data (including other percentiles) relate to the HBM HBGV can be given in the background information of the indicator.

Data were visualized using Excel. The graphs visualise P_50_ and P_95_ as bars and the HBM HBGVs were included as line plots. Alternatively, indicators were plotted against, for instance, time (producing a time-trend) and/or were stratified according to other descriptive parameters (e.g., gender and age), depending on the available information and the policy questions. Visualisation and graphical presentation of the indicators were not thoroughly discussed and lay out may still change.

## 3. Results and Discussion

### 3.1. Selection Criteria for HBM Indicators

Inspired by existing work on HBM and other indicators, a set of criteria for how to select substances to create HBM-indicators was developed (see Table 1). Two main aspects are relevance for policy, society, health, and quality of the collected data (availability, comparability, ease of interpretation). 

The discussion of the experts resulted in the conclusion that an indicator needs to ‘answer’ questions of policy and society. It can be seen as a road sign for policy making. It is a simplification that helps users to absorb information quickly but this also means that it increases the amount of context needed to interpret the information in a correct way. De-contextualisation is one of the most common and yet complex risks in the use of indicators. Selection of exposure to a chemical using HBM data includes weighing off the compounds’ relevance to the availability of qualitative data. 

Overall, during the Copenhagen HBM indicator workshop it was agreed upon that indicators should have policy relevance, data should be available, that exposure burden from a given chemical should be linked to certain health effects, and each indicator should be unique, comparable at international level, and be clear and easy to interpret. However, after the workshop, further discussions led to a consensus in the group that the link to specific health effects would be useful for some but would not be an essential prerequisite for all HBM-indicators. An increase of the concentration of a chemical in a system (human, environment, etc.) may be an early warning signal of a build-up of chemical exposure. 

Two chemical groups, BPA and PFASs, were selected as examples for this paper, going through the process of selection (relevance and data availability) and calculation of the indicators. BPA has a high EU policy and societal relevance [23], mainly due to its endocrine disrupting properties [24]. BPA has been listed as a substance of very high concern (SVHC) in the EU under REACH [25]. Several of the PFASs have been linked to immunotoxic, endocrine, metabolic and possibly carcinogenic effects in humans and in animals. Several REACH and UNEP restrictions are in place for PFASs, including PFOS and its derivatives, for PFOA and its precursors [26], and new evaluations for C9-C14 perfluorocarboxylic acids (PFCAs), for PFHxA, and for short chain PFASs are on-going. Several PFASs are listed by ECHA (European Chemicals Agency) as SVHCs. They occur globally in different environmental media, have a high persistency, and their potential to either accumulate in the environment or bioaccumulate in humans make them of high toxicological and public concern [27]. From the PFAS group, PFOA, PFNA, PFOS, and PFHxS were chosen, representing a group of substances with extremely long half-lives of several years and which have the highest amount of toxicological and epidemiological data of the PFASs. Furthermore, within the context of HBM4EU, for these substance groups, the following policy questions were set out to be answered within the project: (i) Does the current internal exposure differ between countries (which may be possibly linked to different regulations)? (BPA); (ii) Do internal exposure levels (BPA, PFASs) or profiles (PFOS, PFOA) differ by age group?; (iii) Has the internal exposure to PFASs decreased over time (considered as a means in which to check whether policy measures limiting the use of PFOS and PFOA might have worked)? (PFASs). Considering data availability, currently this is ongoing work within HBM4EU. However, for both substance groups, published data on different age groups were available either from six European countries collected within the DEMOCOPHES project (BPA) [13,28], or from two Scandinavian countries (PFASs, [15,16,17,18]).

### 3.2. Construction of HBM Indicators

#### 3.2.1. HBM Indicator of Internal Exposure

From the Copenhagen HBM indicator workshop there was consensus to calculate indicators for P_50_ and P_95_ values. These values are typically reported in scientific studies, and by presenting both, both average and higher-exposed individuals are visible in the graph. The 95th percentile has been selected in many studies as a based percentile to derive reference values (RVs) from HBM studies [29]. From a regulatory perspective, this choice enables a straightforward interpretation of RVs across studies, countries, chemical substances, time, and sub-population groups. 

**Figure 1:**
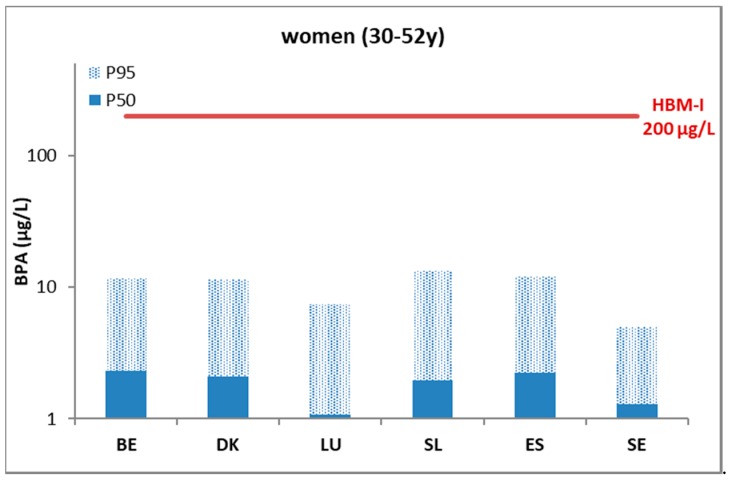
Urinary levels (µg/L) of bisphenol A (BPA)-equivalents in mothers (50th and 95th percentile) in six EU countries (DEMOCOPHES) benchmarked against the German HBM-I guidance value below which no adverse health effects are expected according to current knowledge. BE: Belgium; DK: Denmark; LU: Luxembourg; SL: Slovenia; ES: Spain; SE: Sweden.

**Figure 2:**
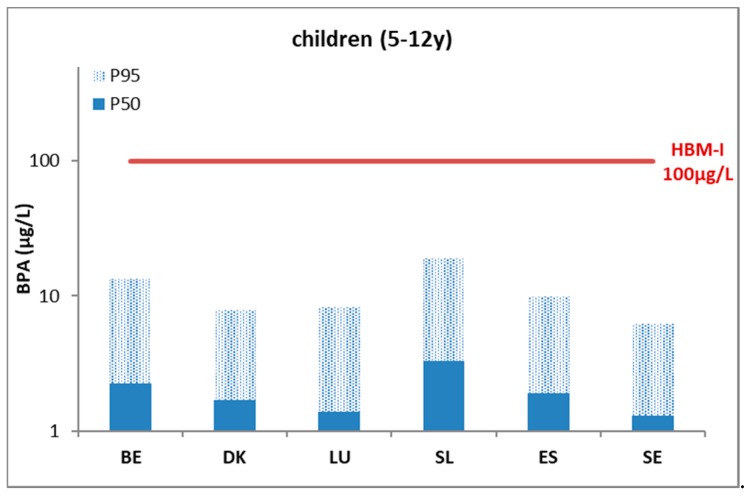
Urinary levels (µg/L) of BPA-equivalents in children (50th and 95th percentile) in six EU countries (DEMOCOPHES) benchmarked against the German HBM-I guidance value below which no adverse health effects are expected according to current knowledge.

For the used data on urinary BPA, Covaci et al. (2015) reported quite similar levels in children and mothers, and the levels in the different countries were close together [13]. They further reported that compared to the European mean, significantly (*p* < 0.05) higher values were observed in children from Slovenia and significantly lower levels were found in Sweden. For the mothers, significantly higher concentrations were observed in Belgium and Denmark, while levels in Sweden and Slovenia were significantly lower [13]. The small differences among the countries can indeed be deduced from the P_50_ country indicators pictured in Figure 1 and Figure 2. For Luxembourg, around 50% of the samples had a value below the LOQ (limit of quantification). The LOQ for Luxembourg was a factor of 5 to 10 higher compared to other countries. 

**Figure 3:**
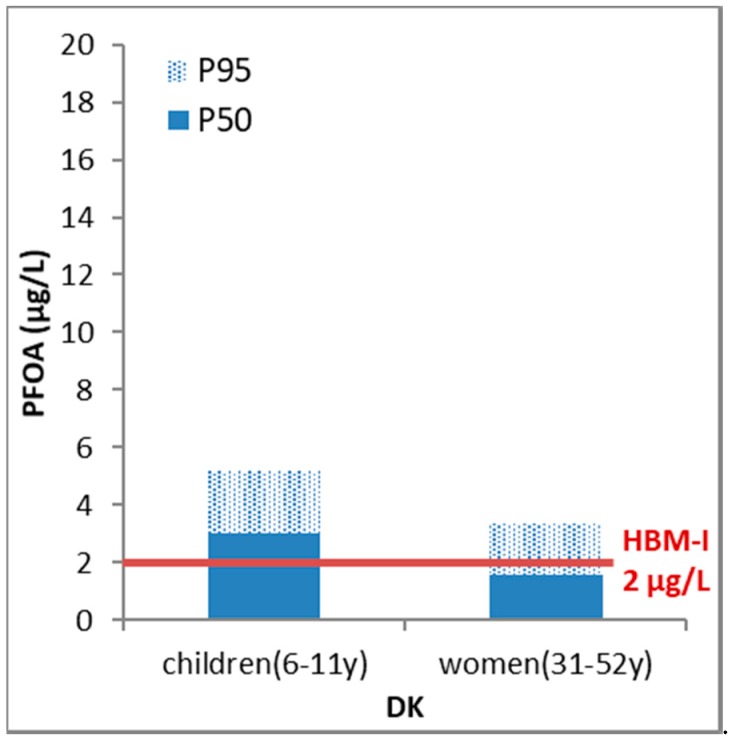
Benchmarking HBM results for perfluorooctanoic acid (PFOA) in women and children (50th and 95th percentile) (DEMOCOPHES) against the German HBM-I guidance value. At concentrations below the HBM-I value no adverse health effects are expected according to current knowledge.

**Figure 4:**
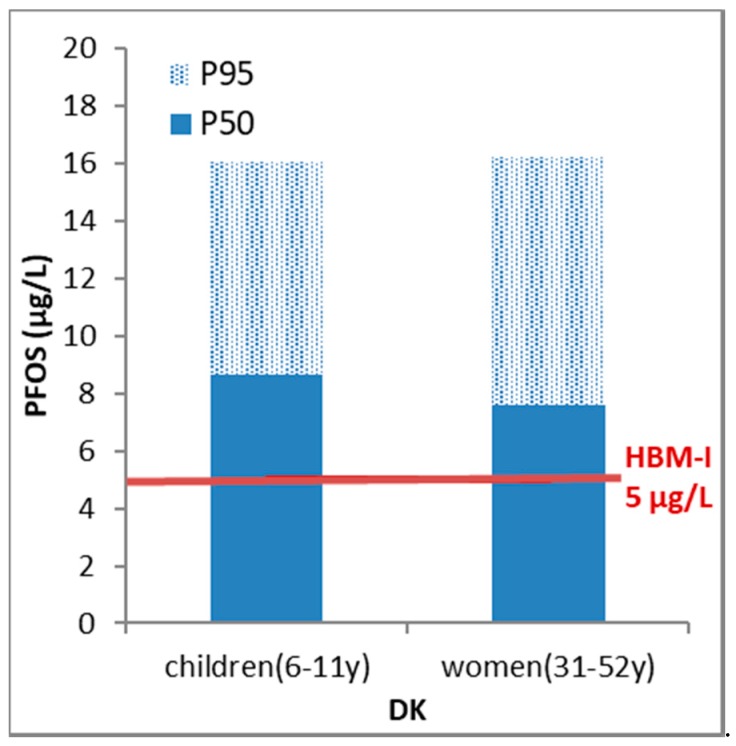
Benchmarking HBM results for perfluorooctanesulfonic acid (PFOS) in women and children (50th and 95th percentile) (DEMOCOPHES) against the German HBM-I guidance value. At concentrations below the HBM-I value no adverse health effects are expected according to current knowledge.

From the DEMOCOPHES study, published PFOS and PFOA data were only available from Denmark. PFOS was more abundant than PFOA in the plasma samples of both mothers (for P_50_, a factor of 4.8 higher) and children (for P_50_, a factor of 2.9 higher). The levels were significantly higher in children compared to their mothers for PFOS (for P_50_, a factor of 1.1) and PFOA (for P_50_, a factor of 1.9) (Figure 3 and Figure 4). This was also the case when adding up the levels (on a molar basis) of both PFOS and PFOA: the sum of P_50_ values expressed on a molar basis was equal to 0.019 µmol/L for mothers and 0.025 µmol/L for their children. It should be noted that for a group of substances, the simplest way is for the substance group indicator to be the sum of HBM exposure levels expressed in, for example, µg/L. A more correct way is indeed adding the molar concentrations of each single substance, as then the high molecular weight substances do not outweigh the others in the total sum. 

**Figure 5:**
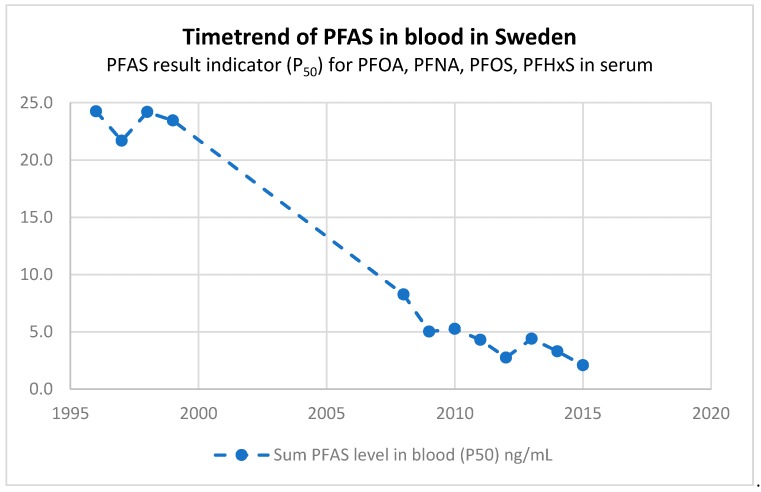
Time trend result indicator of PFOS, PFOA, perfluorononanoic acid (PFNA), and perfluorohexane sulfonate (PFHxS) for the Swedish population (men, women, and children, P_50_). The per- and polyfluoroalkyl substance (PFAS) levels in blood, P_50_ (ng/mL), of PFOA, PFNA, PFOS, and PFHxS were for each year, first summed for each of the age groups, and then averaged over the age groups, resulting in a group substance population indicator. The time trend shows a steady decrease since 1996 for the total PFAS HBM exposure levels, mainly driven by a decrease in PFOS levels. Note: older people were not included for the years 2012–2015.

An alternative indicator for exposure that could be used for a group of substances was calculated from PFOS, PFOA, PFNA, and PFHxS data from the Swedish population (men, women, and children) (Figure 5). This type of indicator, summing up (for each year) the individual compounds in each age group and averaging them over those groups, gives a picture of the combined chemical pressure at the population level. A disadvantage of summing chemical concentrations is that the information on the individual substances may be lost—is the concentration of substance x going up and substance y going down over time? Such information might, however, be put in a layer behind the main indicator, which then could be consulted by experts. The main purpose of such a group result indicator is to identify highly exposed areas or sub-populations and thereby to inform targeted risk management.

Figure 5 shows that for the sum of four PFASs there has been a steady decrease over time since 1996, which mainly is driven by a decrease in PFOS. This indicates that global policy measures to limit the use of PFOA and PFOS, in particular, have worked. It should be noted that indicators need to be accompanied by additional information. Since these four PFASs are highly bioaccumulative and bind to proteins in the blood (half-lives of 3.8–8.5 years), HBM serum levels typically rise with age. The decrease in Figure 5 might be confounded, as older people were not included for the years 2012–2015.

Both the strength and the weakness of a group indicator is that it just simplifies total chemical exposure, which can be followed over time, and which may warrant further investigations or risk management options. The group result indicator has the advantage that it can include substances that have replaced phased-out compounds. This indicator may therefore be used to survey regrettable substitution. Indeed, in the context of PFASs, this feature is very relevant, as in recent years perfluoropolyethers (PFPEs) and their carboxylates and fluorotelomer alcohols and thio-ethers and (polymer) derivatives thereof have substituted many of the PFOA and PFOS uses [30]. They are estimated to be, however, similarly persistent, and, from the little toxicological information available, they may pose many of the characteristics of the chemicals they substituted [31]. 

#### 3.2.2. HBM Indicator for Health Risk

Aside from calculating simply the P_50_ or P_95_, comparison with HBGV allows for an estimation of the adverse health outcome risk. This type of indicator is a subcategory of an environmental performance indicator (EPI). The extent of exceedance (EE) indicator can also be seen as a distance to target indicator or a margin of (internal) exposure indicator [32,33,34]. In the current paper, we used as HBGV the HBM-I values from the German Human Biomonitoring Commission [19]. Other institutions (e.g., US Summit Toxicology [35,36]) may derive other HBGVs (e.g., BE or Biomonitoring Equivalents) for an identical substance. Alternatively, different HBGVs derived for one substance can be applied to display uncertainty on the indicator (see Flemish case: [37]). Showing the existence of several HBM HBGVs for an identical substance can hamper interpretability but increases transparency towards policy makers, risk assessors, and risk managers. For understandability and simplicity reasons, the choice was made to show only one HBM HBGV per substance, which is a normative decision. Another aspect to keep in mind is that revision of the indicators over time can be necessary, since HBM HBGVs may decrease as the chemical becomes better characterized in terms of toxicity. Similar types of indicators were also proposed in the BRIDGE Health project [38,39] and the NORMAN network [40], as it includes an aspect of risk of adverse outcome. Such impact indicators may be used to assess how well implemented policies have worked to reduce health risks in a population, and can also be used to identify highly exposed areas or sub-populations and thereby to inform targeted risk management.

For the results of BPA reported by Covaci et al. (2015) [13], it can be seen from Figure 1 and Figure 2 that the P_50_ and P_95_ values for each country were below the HBM HBGVs. Calculating the percentage of the population with levels above the HBM-I value (PE), this was <5% for the Danish children and the Belgian mothers and children (derivation based on Max. and P_95_ levels). For other countries, this was 0% for both children and mothers. The extent of exceedance indicator (EE), based on the ratio of the 95% percentile over the HBM-I guidance value, was <1. As the outcome of this indicator is largely influenced by the number of samples studied and the percentile selected for comparison, it is informative to provide the value of *N* together with the indicator. The numbers of participants per country of the BPA study are given in Table S2 (supplementary material). 

For the Danish PFOA and PFOS, it was clear from Figure 3 and Figure 4 that a substantial amount of the Danish population exceeded the German HBM-I value. Especially for children, the calculated PE was >50% for both substances. The calculated extent exceedance indicators (EEs) showed that based on P_50_ for PFOA and PFOS the HBM-I value was exceeded 1.5–1.7 times while based on P_95_ for PFOA and PFOS the exceedance was 1.7–3.2 times over the HBM-I value. 

The choice of the percentile P_x_ used to calculate the EE involves a value judgement, which may depend on the policy question, on risk management options, as well as on more scientific issues. A lower percentile, such as P_50_, ‘neglects’ half of the population, which would not be represented in the EE. When using the P_95_ for the calculation, one might, on the other hand, include persons with extreme habits or even with genetic/biological diseases. Altogether, some examples of issues that could be considered in making the choice for a specific (higher or lower) percentile are: chronic character and severity of health outcomes (which also affect the HBM HBGVs); biological half-life in humans, with short-lived metabolites possibly leading to excretion peaks [41]; focus on highly exposed people (e.g., due to occupational exposures or living at contaminated sites); availability of the percentiles needed. It is up to the decision makers to decide if they want to set a limit of exceedance, and in that case, what the limit should be. The choice of where to set the limit of exceedance depends on the level of protection wanted. The chosen protection level, and hence extent of exceedance, is a value judgement, and should be set by policy or decision makers.

This demonstrates clearly that the selection of health-based guidance values and percentiles selected for comparison have a strong normative reference frame. This should be clearly explained in a transparent way, as this is driven by societal values that may shift over time and place.

### 3.3. Uncertainties and Pitfalls Related to Use and Construction of the Indicators 

#### 3.3.1. Precision and Reliability of the Biomarker Measurements

HBM data should be comparable when data from different populations are compared. The biomarkers should be analysed in the same type of sample tissue and chemical analysis of the biosamples should provide comparable results. However, this cannot always be guaranteed currently. In contrast to environmental monitoring or food and feed monitoring, human biomonitoring of the general population is not embedded in a legal framework at EU level. Internal and external quality assessment schemes and standard operating procedures are often available for well-established traditional biomarkers, but they are not obligatory and do not exist for newer biomarkers. Analytical methods may differ among studies, as well as pre- and post-sampling protocols. Sensitivity of analytical methods may differ among labs. This may have implications on the number of samples that are above the limit of quantification (LOQ) and influences the distribution of the reference data of the study population. The use of higher percentiles, such as P_50_ or P_95_, for the indicator are relatively independent of LOQ, whereas lower percentiles of the dose distribution may be influenced by differences in LOQ (e.g., as for BPA in the DEMOCOPHES data). HBM4EU will establish a network of laboratories within Europe that will produce high quality and comparable HBM data for selected priority substances. A stringent quality assurance quality control process is being installed. 

To have a reliable estimate of the percentiles with a reasonable narrow confidence interval, the International Federation of Clinical Chemistry (IFCC) recommends having valid data from at least 120 individuals from the study population [29]. This should be taken in mind if selecting datasets. However, this number of 120 participants is a minimum, as variability in the population characteristics may be high, including differences in age, sex, diet, socio-economic status, and life style, which may confound comparability of the datasets.

#### 3.3.2. Selection and Other Biases

Indicators derived from HBM data are complex because they must take account factors such as the variability in susceptibility in individuals and variability in co-exposures.

To compare HBM datasets from two study populations living, for example, in different geographic areas, one should make sure that the other determinants of exposure in the two study populations are comparable in order to prevent misinterpretation of differences in exposure between the study areas. Statistical models using individual HBM data can adjust for differences in the characteristics of the study populations. This is not possible if the indicator has to be constructed from already aggregated datasets. 

#### 3.3.3. Privacy and Ethical Constraints

The use of individual data has the advantage that they can be stratified and aggregated into population subgroups, such as age and sex or geographical regions. This allows specific research and/or policy questions, relating to, for example, social, economic, and equity issues, to be answered. The use of individual datasets requires, however, that an informed consent has been signed by the individual study persons, and that it covers use of the data for that or a comparable purpose. Compliance is also needed with the General Data Protection Regulation (GDPR, EU Regulation 2016/679) for safe transfer and analysis of the individual data together with other datasets without risking that the natural identity of individuals can be revealed. 

#### 3.3.4. Representativeness of the Target Population

Indicators should be built with data that represent the target population. This requires that the group of interest is sufficiently represented in the study population at the same frequency as it is in the target population. As participation rates differ, minorities and individuals of low socio-economic class are often underrepresented in population studies. Studies might also be designed for specific vulnerable sub-populations, such as pregnant women and children. This introduces a selection bias that, to some extent, can be solved with the use of weighing factors that can compensate for differences in participation rates among groups of interest. 

#### 3.3.5. Uncertainty of Health-Based Guidance Values

As mentioned earlier, health-based guidance values (HBGV) may shift over time as more scientific knowledge and data become available to improve analysis of exposure response associations. Moreover, the discovery of the capacity of certain synthetic chemicals to cause health effects at low-level exposure is ongoing (e.g., for endocrine disruption). The impact of long-term low-level exposure is still a knowledge gap [42]. The derivation of HBGVs depends largely on expert judgement. Different expert groups may provide different opinions even after evaluation of the same datasets. Safe levels for various PFAS substances are still under discussion, for example, in the US [43,44]. In Europe, the European Food Safety Authority (EFSA) has recently (2018) proposed to change the tolerable daily intakes (TDIs) from 2008 of PFOA (1500 ng/kg bw/day) and PFOS (150 ng/kg bw/day) [45] to tolerable weekly intakes (TWIs) of PFOA (6 ng/kg bw/week) and PFOS (13 ng/kg bw/week). This corresponds to lowering by a factor of 1750 for PFOA and 81 for PFOS, respectively [46,47]. The main reasons for this drastic lowering of the tolerable intake is the inclusion of epidemiological studies, where total cholesterol is the leading health effect for PFOS and PFOA. Update of the HBM-1 levels of PFOA and PFOS accordingly may result in a higher proportion of a population exceeding the health based limit values. If HBGVs are used for calculation of the HBM indicator of health risk, at least the source of the HBGV should be clearly mentioned. It may be desirable for transparency reasons to mention or indicate different HBGVs, as they are currently available from different international (research) organisations. This will enhance transparency and will visualise the degree of (un)certainty related to these values. 

#### 3.3.6. Associations between Datasets

Both in the EU and internationally e.g., in the United Nations (UN) and the WHO, there is a wish to improve the evidence base liking environmental quality to human health. HBM exposure and health risk indicators are informative, as they are positioned on the continuum between exposure and health. However, associated trends between HBM indicators and environmental or health indicators should always be interpreted carefully, as such ecological associations do not prove causal linkage. 

### 3.4. HBM Indicators in the European Indicator Field—The Way Forward

#### 3.4.1. Building a Core set of Indicators on the Environmental Exposure and Health Continuum

In the field of environment and health, there are three European Core Indicator lists, namely the Environmental Core Indicators of the European Environmental Agency (EEA) [48,49], the WHO European Region ENHIS (Environment and Health Information System) list [50], and the European Core Health indicators (ECHI) [51]. HBM indicators are currently not part of this. The EEA is one of several EU–level bodies that produce environmental indicators, in cooperation with Eurostat, the Directorate-General (DG) for the Environment and the Joint Research Centre (JRC). At international level, the OECD, the United Nations Statistical Division (UNSD), and United Nations Economic Commission for Europe (UNECE) regularly publish environmental indicators (for which internationally comparable data exist) and/or environmental performance reviews. 

HBM indicators are additional tools to follow specific chemicals in the pressure-state-impact and response framework. They help to provide a more comprehensive view of the fate of exogenous chemicals that enter the human body. It is important for HBM and environmental indicators that the same substance(s) is (are) measured and grouped in the same way. This may be a challenge, because environmental indicators typically are linked to specific policies, which may group chemicals differently. Examples are specifically PFOA or the total organic fluorine in blood, or Tributyltin and/or tin (Sn) in biota. Depending on the substance and its suspected sources and routes of exposure, it would be relevant to compare different types of environmental indicators (e.g., emissions, concentrations in products, etc.). Unfortunately, there is a lack of monitoring and, consequently, indicators lack for chemicals in most articles and products in the EU. IPCHEM, the EU Information Platform for Chemical Monitoring [52], hosts research data and provides links to chemical occurrence data in Europe. It contains four modules for environmental monitoring data, human biomonitoring, food and feed monitoring data, and for products and indoor air, which currently is being populated with existing research data for products. Links are also being set up to other international databases, for example, to the US EPA and the OECD. Over time, IPCHEM may therefore become the tool of choice, providing a comprehensive picture that allows HBM data to be placed next to other datasets, which should help with describing trends in exposures sources and, for example, sub-populations and regions at risk. This information could then be accessed by risk managers to mitigate or prevent future risks of prioritized chemicals. There is the intention to stratify HBM data by not only age, sex, and geographical region but also, for example, socio-economic status (SES). The relevance is connected to its possible link to (health) in-equalities, some of which may be caused by exposure to chemicals, being one of several stressors that decrease peoples’ resilience. There is also an interest from EU agencies to include HBM indicators that could support the measurement of progress on the strategic objective of EU health policies to foster good health and prevent disease [53] and the 7th EAP objectives to reduce impacts of hazardous chemicals on human health and the environment.

#### 3.4.2. Indicators for Mixtures and Hot Spots

In real life, individuals are exposed to complex mixtures of man-made chemicals every day. This is complex, as each individual will be exposed to a different mixture and no simple methods are available to monitor the presence of a mixture in the individual and to assess its impact. Hence no HBM-based indicators for exposure to mixtures are available yet, although the policy demand is clear [54]. The 7th EAP calls amongst others for strategies on mixtures, and for pharmaceuticals in the environment. Information on exposure of EU citizens to mixtures is largely lacking. Most HBM studies measure only biomarkers for a limited set of chemicals and exposure to most chemicals remains undetected. HBM4EU develops methods for broad chemical screening of human samples to address these needs. This information can generate knowledge on (1) exposure and (2) risk, when exposure information is combined with information on hazard. Next to improving monitoring of exposure, there is also the need for mixture risk assessment. Although some guidance and concepts are available for grouping substances and for assessing mixture toxicity [55], a systematic approach and health-based reference limits are still lacking. It is anticipated that when progress is made in this field, the construction of indicators will follow. 

HBM indicators of combined exposure to chemicals can serve as a surveillance tool to identify subgroups of individuals that are highly exposed to multiple chemicals, such as people that live in the vicinity of polluted hot spot locations. This information can be used to prioritise further studies to elucidate, for example, the risk and/or sources of pollutions, which can inform the risk management. The HBM indicator of PFAS is an example of such an indicator. When developing HBM indicators for risk assessment, a concern is whether it can be justified to sum chemicals with different or unknown modes of toxicological action. Furthermore, the potential for synergistic or antagonistic action among the compounds has to be taken into account with a mathematical formulation that would introduce biochemical interaction terms (factors) when constructing the cumulative exposure indicator reported. When information of toxicity is lacking for a chemical, REACH allows the toxicity of a chemical with ‘similar’ structure or properties to be used, which is termed read-across. It remains to be further discussed how read-across can be applied to make HBM indicators for groups of structurally similar chemicals for which there is limited information on modes of actions.

## 4. Conclusions

Human biomonitoring (HBM) indicators are easily interpretable tools used to monitor internal exposure to (a) chemical substances in various dimensions in an absolute sense (temporal scale, spatial scale, exposure levels in subpopulations) or in a relative sense, for example, referred to statistically-derived reference levels in the general population or normalised to some levels regarded as safe such as the HBM HBGV (health- based guidance value). As such, HBM indicators can convey messages to policy makers on: (1) the exposure burden from chemicals, which may help to prioritize and inform risk management and future policy making, and (2) can highlight the success or failure of regulatory actions that have been taken. Consequently, HBM indicators can be used as a governance instrument to deliver information about the progress that has been made in reaching policy objectives, such as minimizing the exposure burden and the health impact of chemicals on vulnerable sub-populations or the whole European population. For observing changes in chemical exposure burden, it is necessary to collect data periodically, which will allow to consider both the current chemical exposure status and to interpret it in the light of the historical time trends. 

As a result of an expert workshop, HBM indicators were developed for exposure to single substances and to a group of substances and for health risk. HBM indicators for exposure are those that take into account the measured exposure, which may or may not be benchmarked against HBM reference values (HBM RVs) for the general population. Time trends, spatial trends, stratification by age and gender, socioeconomic status, etc., can be displayed in relation to the data availability and policy goal, if present. When HBM health-based guidance values (HBGVs) are available, measured exposure can be benchmarked against these and inform risk assessment (HBM indicators for health risk). The percentage of the population exceeding the HBGV (PE) and the extent of exceedance of HBGV (EE) can be calculated. Which percentage (percentile) of the population that is used in calculating the HBGV PE and EE depends of the level of protection wanted, and therefore involves value judgement. As there may be different preferences, the decision would benefit from a societal and political debate, and therefore more information and transparency on the distribution of the individual HBM data should be given.

Overall, the workgroup agreed that the indicator should contain a clear and easy to understand description. The policy questions also largely determine the type and design of the indicator. Generally, the indicator can be expressed in number format or infographics to order to increase the readability. Taken all together, the main aim of HBM indicators is to allow straight forward follow-up of European chemical safety.

Indicators should be seen as communication tools that should be able to close the gap between the science community, chemical risk managers, the public health community, and the public at large. They reduce complexity through their ability to be used in simple messages that should be clear and understandable for a large audience. 

The three angles of HBM indicators—going downstream to describe the health effects of the measured levels of chemicals in humans, going upstream to identify environmental and other sources which can be reduced, and digging deeper to understand if certain groups are at risk—lend themselves well to creating a compelling narrative of chemicals impact on human lives. Throughout the work with indicators and their construction, it is therefore essential to keep in mind whether one representation or another facilitates or hampers the communication to the target audience. The hope is that such discussions will help to raise awareness across Europe and will make more people interested in making smart choices in relation to chemicals [56], whether it be in consideration of which products they buy, or the policies they want to support.

## Figures and Tables

**Table 1:** List of criteria for the selection of chemicals for which human biomonitoring (HBM) indicators can be derived.

**Relevance**	
EU policy relevance	• Public health issue, burden of disease
	• Clear policy question
	• Preparing policy and signalling function (chemicals of concern)
	• Evaluation of policies (implementation)
	• Help investigate potential links between environment and health
	• Clear possibilities for prevention and risk management options
	• Disaggregation possible into population subgroups or areas of particular interest, such as based on regional areas, socio-economic inequity, or vulnerable groups
Societal relevance	• Public demand for more information on a topic
Health relevance	• Evidence of internal exposure
	• An association with adverse health outcomes has been demonstrated (not mandatory)
	• Human biomonitoring health-based guidance values (HBM HBGVs) preferably available (health risk)
**Data Quality**	
Data availability	• HBM data available from European countries
	• Representative HBM data of the target (subpopulation, area, time window) addressed in the policy question
	• At least 120 persons per study population with valid HBM data
	• Time or spatial trends
Comparability	• Availability of standardized HBM analytical method
	• Transparent and scientifically sound
	• Providing benchmark for international comparison
Indicator type	• No overlap/strong correlation with other indicators (e.g., from same source)
	• Interpretability: simple interpretation in relation to policy question, intuitively obvious what the indicator stands for
	• Raise awareness across different stakeholder groups

## Supplementary Materials

Table S1. Frameworks and organisations in which environmental health and HBM indicators are valuable; Table S2. Result and impact indicators based on DEMOCOPHES data for urinary BPA using existing.
