# Supplementary material for: Development of Policy Relevant Human Biomonitoring Indicators for Chemical Exposure in the European Population
Source: Int J Environ Res Public Health. 2018 Sep 21;15(10):2085. doi: 10.3390/ijerph15102085 (PMC6209865; doi:10.3390/ijerph15102085)
Supplement: Supplementary file 1 [file ijerph-15-02085-s001.pdf]

International Journal of
Environmental Research and Public Health

Supplementary material

Table S1. Frameworks and organisations in which environmental health and HBM indicators are valuable.

Domain	Frame/Organisation	Objectives	HBM or HBM indicators included?	Data sources	Policy target
National	US- CDC	Protecting the health and safety of people at home and abroad, providing credible information to enhance health decisions, and promoting health through strong partnerships.	**HBM:** NHANES surveys all over US population<br><br>**HBM indicators:** see ROE indicator below for USEPA	HBM time trends	- Find out which environmental chemicals actually get into people.<br>- Measure how much exposure a person has.<br>- Assess exposure for health studies of certain groups of people such as children or women of childbearing age.<br>- Determine which population groups, such as minorities, people with low incomes, children, or the elderly, are at high risk for exposure and adverse health effects.<br>- Assess the effectiveness of public health interventions.<br>- Monitor trends in exposure levels over time

Domain	Frame/Organisation	Objectives	HBM or HBM indicators included?	Data sources	Policy target
National	USEPA	a.o. protect all Americans from significant risks to human health and the environment where they live, learn and work.	**HBM**: exposure assessment survey, source investigations, occupational investigations, risk characterization, etc.<br>**HBM indicator**: ROE indicators do (a) Allow EPA and the public to assess whether the Agency is succeeding in its overall mission to protect human health and the environment; (b) Provide valuable input to EPA in developing its strategic outlook and priorities.<br><br>- cadmium in blood (https://cfpub.epa.gov/roe/indicator.cfm?i=61)<br>- cotinine in serum (http://cfpub.epa.gov/roe/indicator.cfm?i=26)<br>- lead in blood (http://cfpub.epa.gov/roe/indicator.cfm?i=63)<br>- mercury in blood (http://cfpub.epa.gov/roe/indicator.cfm?i=64)<br>- pops in serum (http://cfpub.epa.gov/roe/indicator.cfm?i=65)<br>- pesticides in urine (http://cfpub.epa.gov/roe/indicator.cfm?i=66)<br>- phthalates in urine (http://cfpub.epa.gov/roe/indicator.cfm?i=67)	HBM time trends	For example, population data on blood lead concentrations associated with adverse health effects provided impetus for the U.S. Environmental Protection Agency's (EPA's) regulatory reduction of lead in gasoline.

Domain	Frame/Organisation	Objectives	HBM or HBM indicators included?	Data sources	Policy target
National	Health Canada	Health Canada is the federal department responsible for helping the people of Canada maintain and improve their health.	**HBM**: Surveys<br><br>**HBM indicators**: CESI indicator is used to measure progress towards Target 4.8: Chemical Management – Reduce risks to Canadians and impacts on the environment and human health posed by releases of harmful substances.<br><br>http://www.ec.gc.ca/dd-sd/default.asp?lang=en&n=CD4179F6-1/#T4.8<br><br>Indicators on:<br>- cadmium in blood<br>- lead in blood<br>- mercury in blood<br>- BPA in urine<br>- PBDE-47 in blood plasma<br>- PFOS in blood plasma<br><br>http://www.ec.gc.ca/indicateurs-indicators/default.asp?lang=en&n=2D28BA64-1	HBM time trends 2007–2013 geometric mean (µg/L in blood or urine)	Aid in assessing the exposure to environmental chemicals and assessing policies to reduce exposure to chemicals for the protection of the health of Canadians.
National	UBA	- to protect and maintain natural resources, also as an act of responsibility towards future generations,<br>- to advance sustainable development,<br>- to promote environmental protection as a matter of course in the thinking and action of everybody.	**HBM**: surveys<br><br>**HBM indicators**: In the studies on data on the environment (2002, 2009), two indicators (blood lead and blood organochloro compounds) are given.<br><br>These indicators were not selected in 2013, 2015 reports.	HBM time trends 1985–2006 geometric mean (µg/L in blood)	HBM provides scientific data for informed decision making by regulators, policy makers, and the general public. Moreover, it allows for the success of reduction measures to be controlled and for areas for priority action to be identified.

Domain	Frame/Organisation	Objectives	HBM or HBM indicators included?	Data sources	Policy target
National	FLEHS	Generating information on the distribution of biomarker values for a large number of environmental pollutants in a representative sample of the Flemish population.<br>Find temporal and spatial patterns.<br>Monitor policy interventions.<br>Follow-up of exposure and effects in hot spots, etc.	**HBM:** surveys<br><br>**HBM indicators**: indicators developed for<br>- As in urine of adolescents[a]<br>- Cd in blood of adolescents[b]<br>- PFOA in cord blood of newborns[c]<br>- PCBs in serum of adolescents[d]<br>- Pb in cord blood of newborns[e]<br>- HCB in serum of adults[f]<br><br>Since April 2017, available on the following website https://www.milieurapport.be/milieuthemas/milieu-gezondheid	HBM time trends (µg/L in blood or urine) and risk analysis	Awareness-raising activities and implementation of measures (pesticides, POPs, asthma and allergies, hot spots, PAHs, cadmium, lead)
Global	WHO, Parma Declaration on Environment and Health 2010	Increase efforts CEHAPE:<br>Goal 1: safe water and sanitation<br>Goal 2: safe environment and healthy diet<br>Goal 3: improved air quality<br>Goal 4: prevent disease from physical, biological, and chemical environment (e.g., endocrine disruptors, bio-accumulating chemicals).	**HBM:** -<br><br>**HBM indicators:** See WHO indicators below on blood lead in children and dioxins in human milk. Other HBM indicators: need for being developed expressed in declaration		Commitment to act.<br>Health promotion in all policies.<br>Supporting environment and health information system. Development of internationally comparable indicators. Contribution to the development of a consistent and rational approach to human biomonitoring as a complementary tool to assist evidence-based public health and environmental measures, including awareness-raising for preventive actions.

Domain	Frame/Organisation	Objectives	HBM or HBM indicators included?	Data sources	Policy target
Global	WHO European Centre for Environment and Health (ECEH)	a.o. coordinating the development of biomonitoring-based indicators for efficient monitoring of Parma Declaration commitments.	**HBM:** Focus on children/pregnant women. List of high priority biomarkers composed.<br><br>**HBM indicators**: ENHIS indicators about blood lead in children and persistent organic pollutants (POPs) in human milk were already implemented. Need for other indicators being developed.<br><br>Dioxins in human milk and blood lead in children: https://gateway.euro.who.int/en/datasets/enhis/<br><br>Other environmental health indicators not using HBM:<br>- PM10 in outdoor air<br>- Exposure to chemical hazards in food<br>- Exposure of children to second-hand tobacco smoke (SHS)	HBM time trend for dioxins 1988–2007 (pg/g fat)<br><br>HBM geometric mean for lead in blood (μg/dL)<br><br>Time trends 1992–2012 for PM10 Annual mean (μg/m³)<br><br>Intake of heavy metals through food in 2004 (μg)<br><br>Time trends of children exposed to SHS 2002–2007 (Percentage)	- develop biomarker selection criteria<br>- include these criteria to identify HBM indicators for inclusion in survey<br>- testing efficiency of approaches for biomonitoring-based surveillance in support of risk reduction measures.
International	OECD - Organisation for Economic Co-operation and Development	a.o. reduce health inequality; consumer product safety aims to improve co-operation amongst jurisdictions; stimulating innovation	**HBM:** use of HBM data, e.g., assessing the risk of chemicals to children's health: an OECD-wide survey<br><br>**HBM indicators:** -		Safe product innovations, safe packaging;<br>Safe circular economy, as Europe is not rich in raw materials; Follow-up clean technologies.
Global	UNEP - United Nations Environment Programme	Coordinates worldwide actions to reduce emissions and exposure levels	**HBM:**<br>- Minamata convention: mercury<br>- Stockholm convention: POPs<br>- blood lead<br><br>**HBM indicators:** -		e.g., Ban on leaded petrol. HBM was instrument in stimulating policy actions and demonstrating their effectiveness.

Domain	Frame/Organisation	Objectives	HBM or HBM indicators included?	Data sources	Policy target
Global	UN Sustainable development goals	a.o.<br>- ensure healthy lives and promote well-being for all all ages: an important target is to substantially reduce the number of deaths and illnesses from pollution-related diseases.<br>- reduce inequality	**HBM: -**<br>**HBM indicators: -**		e.g., Poverty reduction
EU	SOER (EEA) - State and Outlook European Environment – European Environment Agency	Assessment of European environment's status, trends, and prospects, and placement in a global context.	**HBM:** Current activities to streamline existing information on chemicals in the environment, including human biomonitoring data, should improve the knowledge base.<br><br>**HBM indicators:** -		It informs European environmental policy implementation and analyses the opportunities to modify existing policies.
EU	ECHI - European Core Health indicators	Presenting relevant and comparable information on public health at European level	**HBM: -**<br>**HBM indicators:** -<br>Only two environmental health-related indicators available: regular smokers, PM10 in air	Proportion (%) of people reporting to smoke cigarettes daily for 2008<br><br>Annual average of PM10 1998–2012 (μg/m^3)	Consolidate and expand the ECHI indicator system towards a sustainable health monitoring system in Europe.
EU	EU Strategy for a non-toxic environment: Non-toxic products and material cycles	Identify gaps, deficits, and related improvement opportunities in the management of chemicals in material cycles in order to decrease unwanted effects from chemicals, such as toxic emissions or material stream contaminations.	**HBM:** coordinated HBM efforts at EU level should bridge science and policy by generating targeted evidence on human exposure and associated health outcomes to directly address current policy questions.<br><br>**HBM indicators:** -		Consumer protection. Safety first as guiding principle. Avoid contaminations in product chain.<br>Information on actual exposure (bridging gap between real exposure and exposure estimation).
	EU Strategy for a non-toxic environment: Early warning systems (environment, worker, consumer	Set on the hazards posed by chemicals to human health and the environment in an early stage			HBM can provide the first examples needed to give action and be used to assess the exposure to new contaminants in the population (Norden, 2015). The longer it takes to identify a problem with a

Domain	Frame/Organisation		Objectives	HBM or HBM indicators included?	Data sources	Policy target
		protection, and food safety)				chemical, the longer it will continue to be used. Science moves forward and often finds new toxicity (and often exposures) which had not previously been demonstrated. In reality, we are always dealing with 'currently estimated toxicity' (CET). Also, the identification of new emerging contamination issues at levels which are still far below the health effect levels can support pro-active policy actions and prevent adverse public health impacts.
EU	REACH	SVHC s(Substance s of Very High Concern)	Improve the protection of human health and the environment from the risks that can be posed by chemicals, while enhancing the competitiveness of the EU chemicals industry. It also promotes alternative methods for the hazard assessment of substances.	**HBM:** application of HBM data in dossiers (e.g., phthalates, BPA)<br><br>**HBM indicators:** -		Systematic environmental monitoring and surveillance to track presence and to be aware of any build-up. Follow-up of possible phase out of certain SVHCs and substitution. Enhance the application of the substitution principle in the policy context.
		Very persistent chemicals				Given the potentially serious health and environmental problems, screening is necessary (e.g., PFAS).

[a]: https://www.milieurapport.be/milieuthemas/milieu-gezondheid/humane-biomonitoring/blootstelling-aan-arseen-concentraties-in-urine-van-jongeren

[b]: https://www.milieurapport.be/milieuthemas/milieu-gezondheid/humane-biomonitoring/blootstelling-aan-cadmium-concentraties-in-bloed-van-jongeren

[c]: https://www.milieurapport.be/milieuthemas/milieu-gezondheid/humane-biomonitoring/blootstelling-aan-cadmium-concentraties-in-bloed-van-jongeren

[d]: https://www.milieurapport.be/milieuthemas/milieu-gezondheid/humane-biomonitoring/blootstelling-aan-polychloorbifenylen-concentraties-in-serum-van-jongeren

[e]: https://www.milieurapport.be/milieuthemas/milieu-gezondheid/humane-biomonitoring/blootstelling-aan-lood-concentraties-in-navelstrengbloed-van-pasgeborenen

[f]: https://www.milieurapport.be/milieuthemas/milieu-gezondheid/humane-biomonitoring/blootstelling-aan-hexachloorbenzeen-concentraties-in-serum-van-volwassenen

Abbreviations used in table above:

CDC: Centre for Disease Control
CEHAPE: Children's Environment and Health Action Plan for Europe
CET: Current Estimated Toxicity
CESI: Canadian Environmental Sustainability Indicators
ECEH: WHO European Centre for Environment and Health
ECHI: European Core Health indicators
EEA: European Environment Agency
EFSA: European Food Safety Agency
ENHIS: Environment and Health Information System
FLEHS: Flemish Environment and Health Study
NERC: New or Emerging Risks of Chemicals
NHANES: National Health and Nutrition Examination Survey
OECD: Organisation for Economic Co-operation and Development
PAHs: Polycyclic Aromatic Hydrocarbons
PBDE: PentaBromoDiphenyl Ether
PFAS: Per and PolyFluoroAlkyl Substances
PFOS: PerFluoroOctaneSulfonic acid
PM10: Particulate Matter (aerodynamic diameter <10 µm)
POPs: Persistent Organic Pollutants
REACH: Registration, Evaluation, Authorisation and Restriction of Chemicals
ROE: Report On Environment
SOER: State and Outlook on the Environment Report (European Environment Agency)
SVHC: Substance of Very High Concern
UBA: Umweltbundesamt
UN: United Nations
UNEP: United Nations Environment Programme
USEPA: United States of America - Environmental Protection Agency
WHO: World Health Organisation

Table S2. Result and impact indicators based on DEMOCOPHES data for urinary BPA using existing HBM HBGV: German HBM-I value.

Country	Study group	Number of people	AM (µg/L)	GM (µg/L)	P_{50}	P_{95}	MAX	LOQ	%>LOQ	German HBM-I-value (µg/L)	Percentage of population exceeding the German HBM-I	Extent of exceedance (P_{95}/German HBM-I)	References
DEMOCOPHES – 6 countries	mothers	639		1.78 (1.62–1.94)	1.94	11.13	455.62	0.11-1.00	90.5	200	<5[a]	<1	[b]
	children	653		1.97 (1.81–2.15)	1.96	13.14	821.90	0.11-1.00	91.1	100	<5[a]	<1	
Denmark	mothers	145	4 (0.06–106)	2.00 (1.62–2.47)	2.10	11.45	105.84	0.12	90.9	200	0	<1	[b] and [c]
	children	142	9 (0.06–822)	1.87 (1.53–2.29)	1.71	7.9	821.90	0.12	90.8	100	<5[a]	<1	
Belgium	mothers	125		2.55 (2.07–3.15)	2.3	11.63	455.62	0.2	100	200	<5[a]	<1	[b]
	children	125		2.35 (1.92–2.87)	2.27	13.44	445.24	0.2	96.8	100	<5[a]	<1	
Luxembourg	mothers	56		1.63 (1.21–2.19)	<LOQ	7.44	9.78	1	44.6	200	0	<1	
	children	59		1.78 (1.34–2.37)	1.38	8.28	18.54	1	52.5	100	0	<1	
Slovenia	mothers	106		1.37 (1.11–1.69)	1.97	13.35	69.75	0.11	81.7	200	0	<1	
	children	112		2.63 (2.15–3.22)	3.3	18.86	31.43	0.11	92.9	100	0	<1	
Spain	mothers	113		2.04 (1.65–2.52)	2.26	12.15	39.8	0.20	96.5	200	0	<1	
	children	118		1.83 (1.50–2.24)	1.91	9.84	21.6	0.20	95.8	100	0	<1	
Sweden	mothers	96		1.30 (1.05–1.60)	1.29	5.02	6.34	0.15	100	200	0	<1	
	children	97		1.48 (1.21–1.81)	1.31	6.24	32.4	0.15	100	100	0	<1	

AM: arithmetic mean; GM: geometric mean; P: percentile; LOQ: limit of quantification; LOD: limit of detection; MAX: maximum

[a]: based on comparison of the MAX and P_{95} with the German HBM-I-value. When the data distribution is known in detail a more precise number can be given.

[b]: Covaci et al., 2015

[c]: Frederiksen et al., 2013
